# Brain-Specific Biomarkers as Mortality Predictors after Aneurysmal Subarachnoid Haemorrhage

**DOI:** 10.3390/jcm9124117

**Published:** 2020-12-20

**Authors:** Jaroslaw Kedziora, Malgorzata Burzynska, Waldemar Gozdzik, Andrzej Kübler, Agnieszka Uryga, Magdalena Kasprowicz, Barbara Adamik

**Affiliations:** 1 Department of Anesthesiology and Intensive Therapy, Wroclaw Medical University, Borowska 213, 50-556 Wroclaw, Poland; jaroslaw.kedziora@umed.wroc.pl (J.K.); malgorzata.burzynska@umed.wroc.pl (M.B.); waldemar.gozdzik@umed.wroc.pl (W.G.); andrzej.kubler@umed.wroc.pl (A.K.); 2 Department of Biomedical Engineering, Wroclaw University of Science and Technology, 50-370 Wroclaw, Poland; agnieszka.uryga@pwr.edu.pl (A.U.); magdalena.kasprowicz@pwr.edu.pl (M.K.)

**Keywords:** subarachnoid haemorrhage, outcome, S100B protein, enolase, GFAP, brain damage markers, cerebrospinal fluid

## Abstract

Aneurysmal subarachnoid haemorrhage (aSAH) is a serious condition with a high mortality and high permanent disability rate for those who survive the initial haemorrhage. The purpose of this study was to investigate markers specific to the central nervous system as potential in-hospital mortality predictors after aSAH. In patients with an external ventricular drain, enolase, S100B, and GFAP levels were measured in the blood and cerebrospinal fluid (CSF) on days 1, 2, and 3 after aSAH. Compared to survivors, non-survivors showed a significantly higher peak of S100B and enolase levels in the blood (S100B: 5.7 vs. 1.5 ng/mL, *p* = 0.031; enolase: 6.1 vs. 1.4 ng/mL, *p* = 0.011) and the CSF (S100B: 18.3 vs. 0.9 ng/mL, *p* = 0.042; enolase: 109.2 vs. 6.1 ng/mL, *p* = 0.015). Enolase showed the highest level of predictability at 1.8 ng/mL in the blood (AUC of 0.873) and 80.0 ng/mL in the CSF (AUC of 0.889). The predictive ability of S100B was also very good with a threshold of 5.7 ng/mL in the blood (AUC 0.825) and 4.5 ng/mL in the CSF (AUC 0.810). In conclusion, enolase and S100B, but not GFAP, might be suitable as biomarkers for the early prediction of in-hospital mortality after aSAH.

## 1. Introduction

In patients who have suffered brain injury, the concentrations of certain markers in the cerebrospinal fluid (CSF) and blood are correlated with the severity of brain damage and the outcome [1,2,3]. Markers specific to the central nervous system (CNS) have been the focus of research as potential post-injury outcome biomarkers, in particular those derived from neurons and astrocytes [4]. Neuron-specific enolase is a cytoplasmic enzyme of neurons and has no extracranial sources. Therefore, NSE has the potential to be useful as a marker of destructive processes in the central nervous system. Another marker, S100B, is a protein found predominantly in astrocytes, glial and Schwann cells in the CNS. S100B is released after brain injury and high levels of S100B can be detected in a variety of pathological injuries to the CNS, such as subarachnoid haemorrhage (SAH), acute brain injury, traumatic brain injury (TBI), and acute ischemic stroke [5,6]. Glial fibrillary acid protein (GFAP), a cytoskeleton protein, is another promising marker of brain injury. It serves as an intermediate filament in numerous cell types of the CNS, including mature astrocytes. The concentration of GFAP is higher in brain pathologies such as stroke, intracerebral haemorrhage, dementia, and SAH [7].

Aneurysmal subarachnoid haemorrhage (aSAH), which is subarachnoid bleeding resulting from an intracranial aneurysm, is a serious condition with a high mortality rate and high permanent disability rate for those who survive the initial haemorrhage. Previously published studies indicated that aSAH occurs at a relatively young age and has a mortality rate of 8% to 67% [8,9]. Outcome indicators of aSAH evaluate the patient’s condition on admission and most often include age, admission neurologic grade (assessed with the World Federation of Neurological Surgeons grade and Hunt and Hess score) and the appearance of SAH on admission with a computed tomography scan (assessed with the Fisher grade or a similar classification). Failure of cerebral autoregulation and delayed cerebral ischemia also affects the overall prognosis [10]. In addition to clinical parameters and clinical grades, the use of biomarkers after brain injury may be of interest not only for diagnosis and identification of intracranial lesions, but also for assessing severity, treatment effectiveness, and prognosis. An increase in some biomarkers can be detected before the presentation of neurological deterioration [11]. Consequently, patients can be identified earlier in the acute period after aSAH, as a high risk for a bad outcome and death [12]. Patients with a bad prognosis on admission would require long-term intensive care with frequent neurological examinations and continuous monitoring.

Markers specific to the central nervous system, enolase, S100B, and GFAP were the focus of this research as potential outcome predictors after aSAH [13,14]. In the majority of previously published studies, brain biomarkers were assessed in blood samples of patients diagnosed with aSAH, and only a few studies investigated S100B, enolase, and GFAP in the cerebrospinal fluid (CSF). The procedure of collecting CSF in aSAH patients is performed for clinical reasons, such as acute hydrocephalus, severe intraventricular haemorrhage or to measure and treat elevated intracranial pressure [15,16]. It should also be emphasized that a Hunt and Hess grade ≥ 3 and Glasgow Coma Score ≤ 12 are the thresholds for strong consideration of placing an external ventricular drain [15]. Some studies suggested that CSF drainage reduced the vasospasm-related delayed ischemic neurological deficit and improved outcomes. On the other hand, external ventricular drain management influences the rate of complications, such as infection, a ventriculo-peritoneal shunt, CSF leak, or intracranial subdural hygroma or haematoma; moreover, prolongation of the ICU and hospital stay and the deterioration of cognitive outcome were observed in SAH survivors [17,18]. However, in cases when CSF was collected for a clinical reason, measuring the concentration of brain-specific biomarkers in a CSF sample might provide additional valuable information about the degree of brain cell damage, while measuring brain-specific markers in the blood relies on the assumption that the blood-brain barrier has been damaged, allowing proteins to pass from the cerebrospinal fluid into the bloodstream.

It is important to have sufficiently sensitive markers for the brain that can be determined in the CSF and blood. An accurate interpretation of brain biomarkers in relation to predicting mortality in the acute phase of aSAH treatment requires a deeper understanding of the patterns in which they are released. Therefore, the aim of this study was to investigate whether enolase, S100B, and GFAP would be suitable as sensitive biomarkers for the early prediction of in-hospital mortality after aSAH. We analysed biomarker concentrations in the cerebrospinal fluid and blood in aSAH patients who were initially treated with an external ventricular drain and correlated this to patient outcome at hospital discharge.

## 2. Experimental Section

### 2.1. Patient Population

Seventy patients with a diagnosis of spontaneous aSAH were admitted to the Neuro-Intensive Care Unit (ICU) from 2014 to 2017. Patients who were treated with an external ventricular drain and met the inclusion criteria were included in the study.

Inclusion criteria: age ≥ 18 years, an aneurysm in the cerebral arteries as the cause of bleeding confirmed using computer tomography angiography (CTA), magnetic resonance imaging (MRI) or conventional angiography, ICU admission within 24 h after the clinical diagnosis of aSAH, and CSF that was accessible for collection for three consecutive days after the SAH.

Exclusion criteria: previously diagnosed neurological disease, such as previously diagnosed aneurysms, stroke, epilepsy; neurodegenerative diseases such as Parkinson’s disease, dementia, cerebrovascular diseases, motor neuron diseases, and traumatic brain injury.

Cerebrospinal fluid was collected only if the procedure was medically justified and safe for a patient.

**Figure 1:**
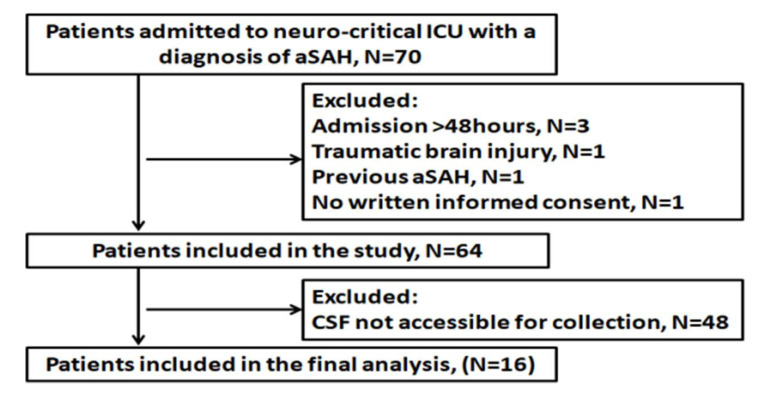
Flow diagram of the study.

Overall, sixteen patients met the inclusion criteria and were included in the final analysis. The study flowchart is shown in Figure 1.

### 2.2. Ethics

The protocol was approved by the Bioethics Committee of the Medical University in Wroclaw (KB–688/2014) and complies with the Declaration of Helsinki of the World Medical Association. In all cases, written informed consent was obtained from the patient or a legally authorized representative.

### 2.3. Clinical Assessment

All patients had an aneurysm in a cerebral artery which had been confirmed as the cause of bleeding by using computer tomography (CT), magnetic resonance imaging (MRI) or conventional angiography. Patients underwent either early clipping or coiling of the detected ruptured aneurysm, within 72 h after the initial bleeding.

The following data were collected: patient age, sex, medical history, aneurysm location, Hunt and Hess, Fisher, WFNS, and GCS grades on admission to the ICU, the timing of surgery or coiling, postoperative complications, the length of stay in the ICU and hospital, and outcomes. A standard clinical treatment protocol was used to manage patients, including early CT angiography and/or three-dimensional digital subtraction angiography, and early surgery when possible, based on aneurysm characteristics, neurological and the general status of the patient. Poor-grade patients with acute hydrocephalus or severe intraventricular haemorrhage were treated with supplementary external ventricular drainage (EVD). Postoperative management included computed tomography of the brain to detect postoperative complications (performed within 24 h after surgical or endovascular treatment), analgesia, sedation, mechanical ventilation, and euvolemia and catecholamine support, where indicated. Induced hypertension was used to improve cerebral circulation and perfusion pressure in patients with cerebral vasospasm (CV) or *delayed* cerebral ischemia (DCI). All patients received nimodipine. Screening for cerebral vasospasm (CVS) was performed daily using neurological examination and transcranial Doppler ultrasound (TCD) measurements. Cerebral vasospasm was defined as a mean cerebral blood flow velocity (CBFV) in the middle cerebral artery that exceeded 120 cm/s or a daily increase of CBFV of approximately 20%.

Focal neurological impairment and weakness or paralysis in the arms and legs were investigated daily by physical examination. DCI was defined as a new focal or global neurological impairment lasting for at least one hour, together with a cerebral infarction that was not caused by other factors (e.g., systemic or surgical complications). On admission, patients were assessed using the Glasgow Coma Scale (GCS). Subarachnoid haemorrhage severity was evaluated with the Hunt and Hess (H-H) scale and defined as severe (4–5) or moderate (1–3). The extent of haemorrhaging was assessed with a computerized tomography (CT) scan of the head and classified using the Fisher scale, where 1 = no subarachnoid (SAH) or intraventricular haemorrhage (IVH) detected, 2 = diffuse thin (<1 mm) SAH, no clots, 3 = localized clots and/or layers of blood >1 mm in thickness, no IVH, and 4 = diffuse or no SAH an intracerebral or intraventricular clot. Outcomes were assessed at discharge from the hospital using the Glasgow Outcome Scale (GOS). Long-term outcomes were assessed with GOS at six months after discharge.

### 2.4. Blood and CSF Sampling and Measurement of Biomarkers

CSF samples (4.5 mL) were collected via an external ventricular drain at the ICU on days 1, 2, and 3. CSF samples were treated with an anticoagulant, centrifuged and the supernatant was aliquoted and kept at −80 degrees Celsius. Blood samples were collected using an intravenous catheter at the ICU on days 1, 2, and 3. Samples were centrifuged, and the supernatant was aliquoted and kept at −80 degrees Celsius. An ELISA assay was used to measure the concentration of S100B (Cloud-Clone Corp., Katy, TX, USA), GFAP (Elabscience, Houston, TX, USA), and enolase (R&D Systems, Minneapolis, MN, USA). The concentrations of the mediators were measured in duplicate with appropriate controls, according to the manufacturer’s instructions using an ELx800 absorbance microplate reader (BioTek, Winooski, VT, USA). As we did not measure CSF biomarkers in another group of neurosurgical patients, we used reference ranges previously reported in the literature. Hajduková et al. established reference ranges for S100B and enolase in CSF based on the results of a study involving a group of 601 patients with no pathological findings (inflammatory, vascular, degenerative, or traumatic impairment of CNS) and without clinical or CT/MRI signs of CNS tissue damage; biochemical, cytological, and immunological values in CSF were normal [19]. The following reference intervals were calculated as 2.5th and 97.5th percentiles: for S100B, 0.3–1.6 ng/mL, and for enolase, 3.5–22.9 ng/mL.

### 2.5. Groups

Patients were categorized based on their status at hospital discharge into two groups: survivors and non-survivors. After 6 months, the clinical status of the patients was assessed with the Glasgow Outcome Scale. To explore the relationship between the CSF and blood biomarkers and outcome, the maximum (peak) values were determined in each patient for the analysed biomarkers, based on values recorded on days 1, 2, and 3.

### 2.6. Statistical Analysis

Continuous variables are presented as the median (interquartile range between the 25th and 75th percentiles); categorical data are presented as numbers and percentages. All analysis was performed with Statistica, version 13 (StatSoft Inc., Tulsa, OK, USA). The distribution was not normal based on the Shapiro–Wilk test. Therefore, statistical analysis was performed using nonparametric tests. The Mann–Whitney U test was used for the comparison of continuous variables between the study groups. For categorical variables, Fisher’s exact test was used to check the level of significance in small sample sizes; contingency tables were used to summarize the relationship between several categorical variables. The Friedman ANOVA test was used to detect differences in biomarker concentrations across multiple test attempts (day 1, 2, and 3). The peak values were determined in each patient for S100B, enolase, and GFAP in the blood and CSF, based on concentrations recorded on days 1, 2, and 3. Spearman’s rank correlation was used to determine the relationship between the CSF and blood biomarkers and the clinical scores. Receiver operating characteristic (ROC) curve analysis was used to measure the ability of the biomarkers measured in the CSF and blood to discriminate between hospital death and survivors by calculating the area under the curve (AUC), including 95% confidence intervals (CI), to determine sensitivity and specificity. A “*p*” value of < 0.05 was accepted as significant.

## 3. Results

Seventy consecutive patients with a diagnosis of aSAH were screened for inclusion/exclusion criteria. Of this number, 64 patients met the inclusion criteria. The 16 patients in whom cerebrospinal fluid was available for collection for three consecutive days after SAH were included in the final analysis. The flow diagram of the study is presented in Figure 1.

The median age was 67 (range 53–76) and 8 patients (50%) were female. On admission to the ICU, 7 patients (44%) were in poor clinical condition (WFNS 4–5). CT scans on admission were graded as follows: 2 patients were graded II (diffuse blood only) on the Fisher scale, 2 were graded III (localized clots and/or vertical layers of blood 1 mm or greater in thickness), and 12 were graded IV (diffuse or no subarachnoid blood with cerebral or ventricular blood). Aneurysms were treated with endovascular coiling in 3 patients and with surgical clipping in 13 patients (81%). During treatment at the ICU, 2 patients showed ventriculitis, which was closely related to site leakage and the duration of catheterization. Both patients received early aggressive treatment, including re-insertion of the drain and treatment with antibiotics. A good outcome was obtained in 1 of these patients; the *ventriculoperitoneal* shunt (*VPS*) was inserted in this patient. The second patient was in poor neurological condition, with an intracerebral hematoma and a blood clot in the ventricles. In both patients, we kept the EVD open with a gradual EVD weaning approach. Attempts were made to close the drain, but due to clinical symptoms such as nausea/vomiting, headache, and altered mental status, the drain was reopened and finally the VPS was inserted. The decision to repeat a clamp trial or place a VPS after a failed clamp trial was made by the attending neurosurgeon.

Nine patients survived and 7 died (44%); all deaths occurred at the ICU. After a 6-month follow-up, the mortality rate had increased to 56% with 2 additional deaths.

GCS on admission to the ICU was significantly lower in non-survivors than in survivors (4 vs. 14 points, *p* = 0.022); the results of other clinical scales (WFNS, Hunt–Hess, and Fisher) did not differ significantly between survivors and non-survivors. The length of stay in the hospital for non-survivors (109 days) was 5× longer than for survivors (21 days). The demographic and clinical data are shown in Table 1.

### 3.1. Blood and CSF Biomarkers Concentration

The peak value is the highest value of the biomarker recorded on days 1 to 3. Non-survivors showed significantly higher peaks of S100B and enolase levels compared to survivors (S100B: 5.7 vs. 1.5 ng/mL, *p* = 0.031; enolase: 6.1 ng/mL vs. 1.4 ng/mL, *p* = 0.011) in the blood. Similar relationships were observed in the CSF: in non-survivors the peak S100B and peak enolase levels were significantly higher compared to survivors (S100B: 18.3 vs. 0.9 ng/mL, *p* = 0.042; enolase: 109.2 vs. 6.1 ng/mL, *p* = 0.015). It is worth noting that, in the CSF, the peak value of S100B and enolase was within the reference range in the group of survivors and exceeded the reference range several times in the group of non-survivors (S100B ref. range: 0.3–1.6 ng/mL; enolase ref. range: 3.5–22.9 ng/mL).

There were no significant differences in the GFAP peak values between the study groups, neither in the blood samples nor the CSF. Detailed data on the peak values of individual biomarkers, including the interquartile range, are presented in Table 2.

**Figure 2:**
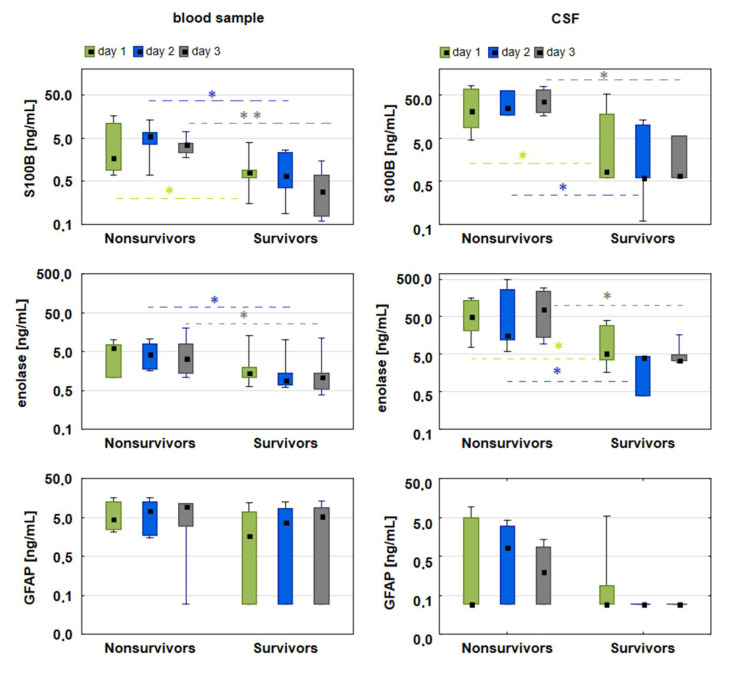
Graphs comparing the levels of S100B, enolase, and GFAP in the blood (**left panel**) and CSF (**right panel**) of non-survivors and survivors. The statistically significant differences in the levels of biomarkers between non-survivors and survivors on corresponding days were marked with * (*p* < 0.05) or ** (*p* < 0.001). A logarithmic scale was used to plot the data. The box plots represent the median values (midpoint) with upper and lower quartiles (box); the whiskers represent the minimum and maximum values.

The biomarker concentrations on consecutive days after the SAH showed significantly higher levels of S100B in non-survivors than in survivors, both in the blood samples (day 1: 1.7 vs. 0.7 ng/mL, *p* = 0.041; day 2: 5.6 vs. 0.7 ng/mL, *p* = 0.007; day 3: 3.6 vs. 0.3 ng/mL *p* < 0.001, in non-survivors vs. survivors, respectively) and the CSF (day 1: 21.9 vs. 3.9 ng/mL, *p* = 0.031; day 2: 25.6 vs. 0.6 ng/mL, *p* = 0.002; day 3: 23.9 vs. 0.6 ng/mL, *p* = 0.002, in non-survivors vs. survivors, respectively). Similar patterns were observed in the changes in enolase concentrations in the blood (day 1: 6.0 vs. 1.3 ng/mL, *p* = 0.046; day 2: 4.2 vs. 0.9 ng/mL, *p* = 0.005; day 3: 3.4 vs. 1.1 ng/mL, *p* = 0.049, in non-survivors vs. survivors, respectively) and the CSF (day 1: 52.6 vs. 5.4 ng/mL, *p* = 0.029; day 2: 16.9 vs. 4.1 ng/mL, *p* = 0.035; day 3: 80.0 vs. 3.4 ng/mL, *p* = 0.031, in non-survivors vs. survivors, respectively). It is worth noting, that on all days, the median of both biomarkers was higher in the cerebrospinal fluid than in the blood. In contrast, the concentrations of GFAP were not statistically different for non-survivors and survivors (Figure 2). A log scale was used to plot the data in order to compare the concentrations using the same range of scales for individual biomarkers in the blood and cerebrospinal fluid. The Friedman ANOVA test did not detect significant differences in the biomarker concentrations between days 1, 2, and 3.

### 3.2. Association between Biomarkers and Severity Scores

The peak concentrations of enolase recorded in the CSF were strongly correlated with GCS (R = 0.7, *p* = 0.005), the Hunt–Hess score (R = 0.6, *p* = 0.027), the Fisher score (R = 0.6, *p* = 0.009), and the WFNF score (R = 0.7, *p* = 0.003) calculated on admission to the ICU. The peak concentrations of enolase in the blood and the peak concentrations of S100B in the blood or CSF did not show a significant correlation to the clinical scores.

### 3.3. Biomarkers as Outcome Predictors

**Figure 3:**
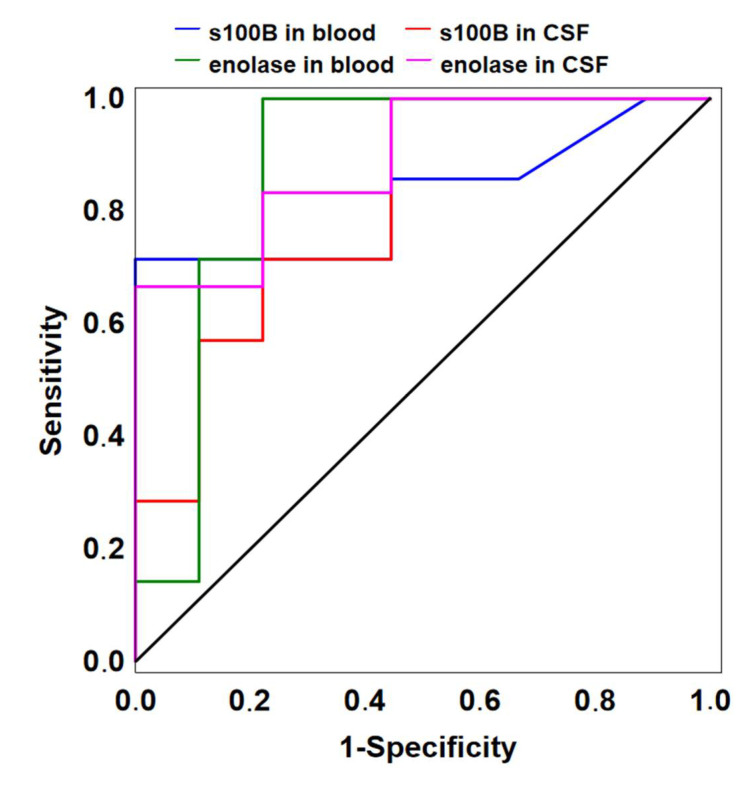
Receiver operating characteristic curve analysis of the peak concentration of S100B in blood (AUC = 0.825) and CSF (AUC = 0.810) and enolase in blood (AUC = 0.873) and CSF (AUC = 0.889) as mortality predictors after aSAH.

In the ROC curve analysis, the peak concentrations of enolase recorded in the blood (AUC 0.873; 95% CI 0.682–1.000, *p* = 0.001) and the CSF (AUC 0.889; 95% CI 0.716–1.000, *p* < 0.001) showed the highest ability to predict hospital mortality (Figure 3). The optimal threshold value for the peak concentration of enolase in the blood was 1.8 ng/mL, with sensitivity of 100% and specificity of 78% and in the CSF it was 80.0 ng/mL, with sensitivity of 67% and specificity of 100%. The peak concentrations of S100B in the blood (AUC 0.825; 95% CI 0.592–1.000, *p* = 0.006; CSF) and the CSF (AUC 0.810; 95% CI 0.595–1.000, *p* < 0.004) also had significant predictive value. The optimal threshold value for the peak concentration of S100B in the blood was 5.7 ng/mL, with sensitivity of 71% and specificity of 100%, and in the CSF it was 4.5 ng/mL, with sensitivity of 100% and specificity of 56%. The GFAP prediction value was not significant either in the blood (*p* = 0.488) or CSF (*p* = 0.443). For comparison, among clinical scores calculated on admission to the ICU, only GCS showed the ability to predict hospital mortality (AUC 0.833; 95% CI 0.632–1.000, *p* = 0.001), while the Hunt–Hess grade, the Fisher grade, and the WFNS score results of the ROC curve analysis were not significant. The optimal threshold value for the GCS was 13 points, with sensitivity of 100% and specificity of 55%.

## 4. Discussion

The results of this study are consistent with the hypothesis that enolase and S100B measured in the cerebrospinal fluid and blood are sensitive biomarkers for predicting in-hospital mortality in patients with aSAH. This study focused on the first 3 days after SAH. We found that S100B and enolase were significantly higher in patients who died than in those who survived, and the peak concentrations of these biomarkers recorded in the cerebrospinal fluid and blood showed a clear ability to predict in-hospital mortality. The authors found very few published reports evaluating the usefulness of cerebrospinal fluid and blood biomarker measurements in predicting mortality in the acute period after aSAH, i.e., at hospital discharge.

Brain cells are damaged to varying degrees by SAH. As a result, various proteins are released into the subarachnoid space from damaged neurons, oligodendrocytes and glial cells [20]. Depending on their molecular weight and the integrity of the blood–brain barrier, these proteins pass to the subarachnoid space and/or to the bloodstream. Proteins that are not physiologically present in the cerebrospinal fluid or blood, or are present at a very low level, can be indicators of brain damage. Here, we showed that determining the concentration of S100B and enolase in the CSF and blood samples could support the early prediction of mortality after aSAH; the concentrations of S100B and enolase measured in the CSF in the early phase of treatment were several times higher in non-survivors than in survivors. Moreover, a similar pattern was observed in the blood samples, indicating damage to the blood–brain barrier, showing that SAH disrupted the integrity of the blood-brain barrier and resulted in the leakage of endogenous proteins. In a previous study by Kellerman et al., when assessing the prognostic value of early blood and CSF concentrations of S100B after subarachnoid haemorrhage, significant differences were found between patients with a bad and good outcome [13]. S100B concentrations in the CSF and blood were significantly higher in patients with an unfavourable outcome (GOS 1–3) in comparison to patients with a good outcome (GOS 4–5), and similar to our results, the concentration of S100B in the CSF was markedly higher than in the blood samples on corresponding days. In a study by Petzold et al., S100B concentrations in the blood measured on admission to the ICU and then at day 1 were significantly higher in patients with a fatal outcome compared with survivors [11]. Importantly, early S100B measurements predicted patient mortality 3–4 days before high intracranial pressure (ICP) readings predicted the same. Quite the opposite results were documented recently by Kiiski et al., who found that elevated S100B at day 1 was strongly associated with a less severe initial clinical representation of aSAH, and the study concluded that plasmatic biomarkers should not be used to guide clinical decision-making in patients with aSAH, in prognostication based on S100B [3].

The time course of blood–brain barrier disruption after SAH has not been fully investigated. The results of both experimental and clinical studies indicate that it is most useful to monitor the biomarkers of brain damage in the first 2–3 days after SAH [21,22]. In our study, the longitudinal profile of biomarkers was rather constant in both non-survivors and survivors over the 3 days of observation. Previously, the time course of barrier breakdown was assessed in an animal model of SAH using Evan’s blue dye extravasation [21]. The breakdown to the barrier began at 36 h, peaked at 48 h, and resolved 3 days after the SAH. In a clinical study by Petzold et al., non-survivors maintained high levels of S100B for over 24 h, slowly decreased on day 2 and reached a plateau on days 3–6 [11]. Similarly, in a recent study by Balanca, a significant decline in S100B in the blood was noted on day 3 after an aSAH diagnosis [22]. The decrease in blood levels of S100B found in several studies suggests that there may be renal clearance into the urine.

The 28-day mortality of patients with SAH was reported to range from 26% to 40% and half of those who survived sustained irreversible brain damage [23,24]. Patients with SAH often have loss of consciousness from temporary global ischaemia, are often sedated and require respiratory support, so clinical symptoms and the calculation of clinical scores may have a limited role in predicting in-hospital mortality. The complex pathophysiology of SAH indicates the need for additional prognostic tools that can help identify patients at high risk of death. In addition to assessing the clinical status of patients through scoring, measuring biomarkers of brain damage in the acute phase aSAH could help predict a high risk of death, especially in unconscious patients. Using biomarkers may improve our ability to distinguish between those who are at risk of death and those who are not, which may affect the treatment strategy. In a study by Rodrigues et al., the results of calculated clinical scores on admission with the WFNS (I–II and II–IV) and Fisher scales (I–II and II–IV) were similarly distributed between survivors and non-survivors and were not associated with in-hospital mortality [25]. In our study, among several clinical scores calculated on admission to the ICU, only GCS was significantly lower in patients who ultimately died compared to those who survived (4 vs. 14 pts.). Other grades (WFNS, H-H, and Fisher) did not differ significantly between survivors and non-survivors. In addition, the ROC probability curve indicated that only GCS could distinguish between survivors and non-survivors, while the results of the WFNS, H-H, and Fisher grades were not significant. Therefore, measuring brain markers in the cerebrospinal fluid and blood could be an additional tool to help identify patients at high risk of in-hospital death after aSAH. In our study, the receiver operating characteristic curve analysis was used to evaluate the suitability of the studied biomarkers for classifying patient outcome, and the Youden index was used as a direct measure of the maximum diagnostic accuracy that each biomarker could achieve. The peak concentrations of enolase showed the highest performance at 1.8 ng/mL threshold in the blood (AUC of 0.873) and at 80.0 ng/mL in the CSF (AUC of 0.889). The predictive ability of the S100B peaks was also very good, with a threshold of 5.7 ng/mL in the blood (AUC 0.825) and 4.5 ng/mL in the CSF (AUC 0.810). For comparison, the clinical scores calculated on admission did not distinguish between a bad and good outcome at hospital discharge, except for GCS, which predicted hospital mortality with high probability (AUC 0.833) and an optimal threshold of 13 pts. There is controversy in the current literature about S-100B and enolase threshold values for predicting an unfavourable outcome or death after aSAH. In a study by Oertel et al., average S-100B values in the blood were significantly higher in patients who died than those who survived and all patients with S-100B > 1 ng/mL experienced an unfavourable outcome (GOS 2–3) or death [26]. In the same study, the outcome was not related to the enolase concentration, contrary to our results. In another study by Kellerman et al., 100% mortality was reported with blood S100B values > 0.7 ng/mL after SAH, but the same relationship was not reported for values in the CSF [13].

In patients with aSAH, both lumbar (LD) and external ventricular (EVD) drains are used for CSF drainage. The main indications for EVD are acute hydrocephalus and intracranial hypertension. EVD drainage might result in drainage-related complications and the total incidence of drainage complications was reported to be approximately 5.3% [27]. These complications include ventriculitis or meningitis, headache due to the ICP drop, intracerebral haemorrhage, deterioration of neurological functions, the need for *ventriculoperitoneal* shunting, and a prolonged stay in the ICU. Lumbar drainage appears to be safer and more effective in removing blood clots from the subarachnoid space, but it is more commonly used in patients with better neurological status [28]. Another problem is EVD management: whether the EVD should be kept open to drainage or only open when needed. It is believed that continuous drainage with low ICP is associated with more complications, including the need for ventricular VPS insertion. Continuous drainage and low ICP may preclude reaching the pressure gradient necessary to restore the natural drainage pathways of the CSF [29]. Rapid EVD wean and intermittent CSF drainage is safe, reduces complications, and shortens the ICU and hospital length of stay as compared to gradual weaning and continuous drainage. In the Qian et al. study, it was found that CSF drainage reduced the incidence of both angiographic and symptomatic cerebral vasoconstriction and significantly reduced the incidence of DIND and cerebral infarction [27]. In addition, the group with CSF drainage had better long-term outcomes and a lower mortality rate. Further work is needed to answer the question of whether there are differences in the results obtained with ventricular and lumbar drainage. The results of previous studies indicate statistically significant differences in white blood cell counts, glucose, and total protein concentrations in the cranial and spinal fluid samples, which may suggest that there may also be differences in biomarker levels [30]. Moreover, the presence of blood clots in the intracranial space may disturb the dynamics of CSF circulation and affect the levels of biomarkers [28].

There are certain limitations to our study. Routine CSF examination only to predict prognosis is impractical due to the invasiveness of the CSF collection procedure. The limitation of this study is the lack of availability of CSF in all patients treated for aSAH, and thus the small size of the study sample. However, it should be emphasized that drainage of the CSF is one of the methods of treating patients with aSAH. About 7–65% of patients with aSAH require placement of EVD to facilitate CSF drainage [31]. Fluid drainage is used to relieve acute hydrocephalus, measure and treat elevated intracranial pressure, and to remove blood from the subarachnoid space [15]. Blood clots in the subarachnoid space are among the pathogenic mechanisms of vasoconstriction and the development of delayed ischemic neurological deficit (DIND), which is the main cause of poor treatment outcomes after aSAH [32]. In some studies, CSF drainage reduced the DIND associated with vasoconstriction and improved treatment outcomes. In our ICU, the procedure of collecting CSF in aSAH patients is performed for clinical reasons only. In patients with CSF drainage, measuring the concentration of S100B and enolase in a fluid sample may provide valuable additional information about the degree of brain damage in the period of early brain injury following aSAH and its relationship to secondary changes such as cerebral vasospasm or the formation of secondary ischemic changes. In addition, due to the small sample size, the impact of the type of intervention (surgical clipping or endovascular coiling) on the release of biomarkers and the prediction of in-hospital mortality after aSAH could not be analysed. Our study was retrospective and conducted in a single centre, which presents a challenge to generalizing our findings. These are preliminary data with interesting results that should encourage further patient recruitment.

## 5. Conclusions

As our study and several previous studies have shown that brain biomarker levels correlate with the clinical outcome of patients after aSAH, these results could be helpful in guiding families and physicians in choosing the most appropriate treatment and comfort measures. However, the clinical utility of biomarker testing will require more consistent and reliable information that indicates what the threshold for a bad outcome would really be. Exact thresholds for increases in enolase and S100B that could predict death after aSAH are still in development.

## Figures and Tables

**Table 1:** Characteristics of patients with aneurysmal subarachnoid haemorrhage (aSAH).

	Total	Non-Survivors	Survivors	*p*
	(*N* = 16)	(*N* = 7)	(*N* = 9)	
Age, [years]	67 (53–76)	75 (38–82)	67 (56–67)	0.606
Gender male/female, [*n*]	8/8	3/4	4/5	0.500
Glasgow Coma Scale	11 (4–14)	4 (4–12)	14 (10–14)	0.022
WFNS, severity of symptoms, [*n* (%)]				0.141
I-III	7 (44)	1	6	
IV-V	9 (56)	6	3	
Hunt–Hess grade, severity of symptoms, [*n* (%)]				0.216
Mild headache	2 (12)	0	2	
Severe headache	2 (12)	1	1	
Lethargy or confusion	2 (12)	0	2	
Stupor	3 (19)	2	1	
Coma	7 (44)	4	3	
Fisher grade, SAH on CT scan, [*n* (%)]				0.411
none	0			
diffuse only	2 (12)	1	1	
clot or thick layer	2 (12)	0	2	
diffuse or none, with cerebral/ventricular blood	12 (75)	6	6	
Delayed cerebral vasospasm, [*n* (%)]	11(69)	5	6	0.634
Intensive Care Unit LOS [day]	15 (8–21)	13 (8–18)	19 (13–28)	0.210
Hospital LOS [day]	43 (20–109)	109 (23–114)	21 (18–57)	0.055

Data are presented as the median (25th–75th percentiles), unless otherwise stated; SAH, subarachnoid haemorrhage; LOS, length of stay.

**Table 2:** The peak values determined in each patient for S100B, enolase, and GFAP in the blood and CSF based on values measured on days 1, 2, and 3.

	Blood			CSF		
	Non-Survivors	Survivors	*p*	Non-Survivors	Survivors	*p*
S100B [ng/mL]	5.7 (1.6–11.2)	1.5 (0.7–2.3)	0.031	18.3 (8.9–78.1)	0.9 (0.6–13.4)	0.042
Enolase [ng/mL]	6.1 (1.9–10.8)	1.4 (1.4–1.6)	0.011	109.2 (31.7–269.0)	6.1 (4.8–16.5)	0.015
GFAP [ng/mL]	4.4 (3.0–15.6)	4.5 (2.3–9.1)	0.469	1.37 (0.03–4.88)	0.05 (0.03–0.09)	0.438

Data are presented as the median (25th–75th percentiles); *p*-value, the difference between non-survivors and survivors.
